# Rare-earth control of phase transitions in infinite-layer nickelates

**DOI:** 10.1093/pnasnexus/pgad108

**Published:** 2023-03-29

**Authors:** Yajun Zhang, Jingtong Zhang, Xu He, Jie Wang, Philippe Ghosez

**Affiliations:** Key Laboratory of Mechanics on Disaster and Environment in Western China Attached to The Ministry of Education of China, Lanzhou University, Lanzhou 730000 Gansu, China; Department of Mechanics and Engineering Science, College of Civil Engineering and Mechanics, Lanzhou University, Lanzhou 730000 Gansu, China; Theoretical Materials Physics, Q-MAT, CESAM, Université de Liège, B-4000 Liège, Belgium; Department of Engineering Mechanics and Key Laboratory of Soft Machines and Smart Devices of Zhejiang Province, Zhejiang University, 38 Zheda Road, Hangzhou 310027, China; Zhejiang Laboratory, Hangzhou, 311100 Zhejiang, China; Theoretical Materials Physics, Q-MAT, CESAM, Université de Liège, B-4000 Liège, Belgium; Department of Engineering Mechanics and Key Laboratory of Soft Machines and Smart Devices of Zhejiang Province, Zhejiang University, 38 Zheda Road, Hangzhou 310027, China; Zhejiang Laboratory, Hangzhou, 311100 Zhejiang, China; Theoretical Materials Physics, Q-MAT, CESAM, Université de Liège, B-4000 Liège, Belgium

**Keywords:** infinite-layer nickelates, rare-earth control, magnetic dimensionality, phase transitions, phase diagram

## Abstract

Perovskite nickelates *R*NiO_3_ (*R* = rare-earth ion) exhibit complex rare-earth ion dependent phase diagram and high tunability of various appealing properties. Here, combining first- and finite-temperature second-principles calculations, we explicitly demonstrate that the superior merits of the interplay among lattice, electron, and spin degrees of freedom can be passed to *R*NiO_2_, which recently gained significant interest as superconductors. We unveil that decreasing the rare-earth size directly modulates the structural, electronic, and magnetic properties and naturally groups infinite-layer nickelates into two categories in terms of the Fermi surface and magnetic dimensionality: compounds with large rare-earth sizes (La, Pr) closely resemble the key properties of CaCuO_2_, showing quasi-two-dimensional (2D) antiferromagnetic (AFM) correlations and strongly localized $d_{x^{2}-y^{2}}$ orbitals around the Fermi level; the compounds with small rare-earth sizes (Nd–Lu) are highly analogous to ferropnictides, showing three-dimensional (3D) magnetic dimensionality and strong $k_{z}$ dispersion of $d_{3z^{2}-r^{2}}$ electrons at the Fermi level. Additionally, we highlight that *R*NiO_2_ with *R* = Nd–Lu exhibit on cooling a structural transition with the appearance of oxygen rotation motion, which is softened by the reduction of rare-earth size and enhanced by spin-rotation couplings. The rare-earth control of $k_{z}$ dispersion and structural phase transition might be the key factors differentiating the distinct upper critical field and resistivity in different compounds. The established original phase diagram summarizing the temperature and rare-earth controlled structural, electronic, and magnetic transitions in *R*NiO_2_ compounds provides rich structural and chemical flexibility to tailor the superconducting property.

**Significance Statement:**

The discovery of superconductivity in *R*NiO_2_ infinite-layer nickelate compounds has recently generated a huge interest. Strikingly, the fundamental physics are strongly rare-earth dependent. Here, a general lattice–electron–spin relationship is established through first- and second-principles techniques. We unveil that reducing rare-earth size softens in-plane rotation, controls the crystal field splitting, and tunes the competition between strongly localized Ni $d_{x^{2}-y^{2}}$ bands and itinerant Ni $d_{3z^{2}-r^{2}}$ bands, which divides infinite-layer nickelates into two groups with distinct Fermi surface, $k_{z}$ dispersion, and magnetic dimensionality. We further bridges different communities by making an explicit comparison with high-T_c_ cuprate and iron superconductor. A complete temperature-dependent phase diagram of this emergent family of compounds and an unified discussion of their structural, electronic, and magnetic properties are achieved.

Infinite-layer nickelates *R*NiO_2_ (*R* = rare-earth ion) attracted increased interest since the recent discovery of superconductivity (1, 2). The observation of superconductivity in this family of $d^{9}$ compounds provides indeed an exciting new platform to explore the physics of high-T_c_ superconductors and has boosted numerous theoretical and experimental studies (1–43).

Comparing with the *R*NiO_3_ perovskites, *R*NiO_2_ can be viewed as derivatives with the removal of the apical oxygen atoms. In *R*NiO_3_, due to the complex interplay among charge, orbital, spin, and lattice degrees of freedom, compounds with different rare-earth ion exhibit quite distinct behaviors and properties (44, 45). As $r_{R}$ decreases, the antiferrodistortive (AFD) rotation pattern changes from $a^{-}a^{-}a^{-}$ to $a^{-}a^{-}c^{+}$ from La to Pr–Lu, and the rotation amplitudes notably increase. The magnetic order undergoes paramagnetic (PM) to antiferromagnetic (AFM) transition, and the Néel temperature first increases and then decreases continuously with decreasing $r_{R}$. In terms of electronic properties, with the decrease of $r_{R}$, the bond disproportionation becomes stronger and there is a transition from the metallic to insulating phase and the band gap increases smoothly (44, 45).

Interestingly, in the *R*NiO_2_ family, depending on the *R*-site ion, there is also a large diversity of their properties. For example, the magnitude and anisotropy of the superconducting upper critical magnetic field (H${}_{c2}$) in LaNiO_2_, PrNiO_2_, and NdNiO_2_ are strikingly different. LaNiO_2_ and PrNiO_2_ exhibit strong anisotropy of the upper critical field similar with that of cuprates (39). However, an unexpected isotropy of H${}_{c2}$ is observed in NdNiO_2_ (5, 6, 39). This behavior is at odds with that of cuprates, but analogous to that of ferropnictides. In the latter, the isotropic H${}_{c2}$ has been related to the $k_{z}$ dispersion of bands at the Fermi surface (46). Note that ferropnictides with three-dimensional (3D) magnetic couplings are multiband systems with both Mott-localized and itinerant electrons and exhibit a 3D Fermi surface. In contrast, Mott-insulating cuprates with two-dimensional (2D) magnetic couplings exhibit single-band character. These suggest that rare-earth control of electronic transition across the *R*NiO_2_ family might be the key factor for the strong difference in H${}_{c2}$.

On the other hand, striking difference also exists in the temperature-dependent resistivity. An unusual upturn of resistivity is observed in NdNiO_2_ at low temperature (1), which is not obvious in LaNiO_2_. It is known that superconducting LaOFeAs (47) and Sr_3_Rh_4_Sn${}_{13}$ (48) also exhibit temperature-dependent resistivity anomaly, both are driven by the structural phase transition. In related *R*NiO_3_ perovskites, AFD rotation of the NiO_6_ octahedra typically reduces orbital hybridization and localizes electrons, resulting in enhanced resistivity, band gap, and metal-insulator transition temperature (45, 49). NdNiO_2_ can be seen as a defective NdNiO_3_ perovskite with missing apical oxygen atoms. Although oxygen rotation motion has never been previously reported in infinite layer NdNiO_2_, it might be questioned if rotation of NiO_4_ squares could eventually induce the anomaly of resistivity.

More broadly, investigations on *R*NiO_2_ compounds primarily focused so far on the electronic and magnetic properties of the high-symmetry P4/mmm phase, while lattice effects and the interplay between different degrees of freedom received much less attention. In related *R*NiO_3_ perovskites, recent works highlighted the strong interplay between electronic and structural degrees of freedom (50–53). Mercy et al. (50) pointed out that the breathing distortions and metal-insulator transition are triggered by AFD oxygen rotation motions. Consistently with that, tuning of the electronic properties from the control of AFD motions has been realized in heterostructures (54). Identifying the fundamental role of *R*-site cation and achieving a more global description of the interplay among structural, electronic, and magnetic properties of *R*NiO_2_ compounds would facilitate the full optimization of their superconducting properties.

In this work, combining first-principles calculations at zero Kelvin and second-principles calculations at finite-temperature, the interplay among lattice, electron, and spin degrees of freedom in *R*NiO_2_ compounds is investigated to provide a consistent model to explore the ground state properties, reconcile different experimental observations, and hopefully disentangle the origin of different behaviors in the infinite-layer nickelates.

From lattice dynamic analysis, our calculations first reveal the presence of out-of-phase rotation motion of NiO_4_ squares in *R*NiO_2_ (*R* = Nd–Lu), reminiscent of the rotations of BX_6_ octahedra in ABX_3_ perovskites, where A is alkaline-earth or rare-earth element, B is transition metal element, and X (chalcogenide, oxide, and halide) is an anion. From this, the microscopic origin of the abrupt upturn of resistivity in NdNiO_2_ is rationalized.

We further reveal that the *R* cation has profound impacts on the Fermi surface and magnetic dimensionality, thereby yielding *R*NiO_2_ into two possible states: (i) one with marked electronic and magnetic similarities with quasi-2D AFM CaCuO_2_ for *R* = La–Pr with $d_{x^{2}-y^{2}}$ bands at the Fermi surface, and (ii) another state with a striking resemblance in the magnetic dimensionality with iron superconductors for *R* = Nd–Lu with $d_{3z^{2}-r^{2}}$ bands occupying the Fermi energy. The direct links among geometrical effect, electronic structure, and magnetic order are established. We suggest that the *R*-site cation-tuned strength of the out-of-plane Ni $d_{3z^{2}-r^{2}}$ band dispersion in La (Pr)NiO_2_ and NdNiO_2_ are likely responsible for their strong difference in the anisotropy of H${}_{c2}$. Eventually, we demonstrate that as in *R*NiO_3_ perovskites, rare-earth ion controlled structural, electronic, and magnetic properties can give rise in *R*NiO_2_ compounds to a rich phase diagram, which offers a unique opportunity to control desired properties by external strategy.

## Results

Rare-earth ion substitution in perovskites and related compounds is well known as an efficient parameter for tuning phase transitions and has become an essential factor to differentiate the behaviors of different compounds within the same family like in *R*NiO_3_ (44, 45) and the investigated *R*NiO_2_ systems (1, 39). Achieving a deeper understanding of the variation of ground state properties across the *R*NiO_2_ series would be a crucial step for rationalizing the exact roles of rare-earth ion.

### Rare-earth control of structural transition

**Fig. 1.**
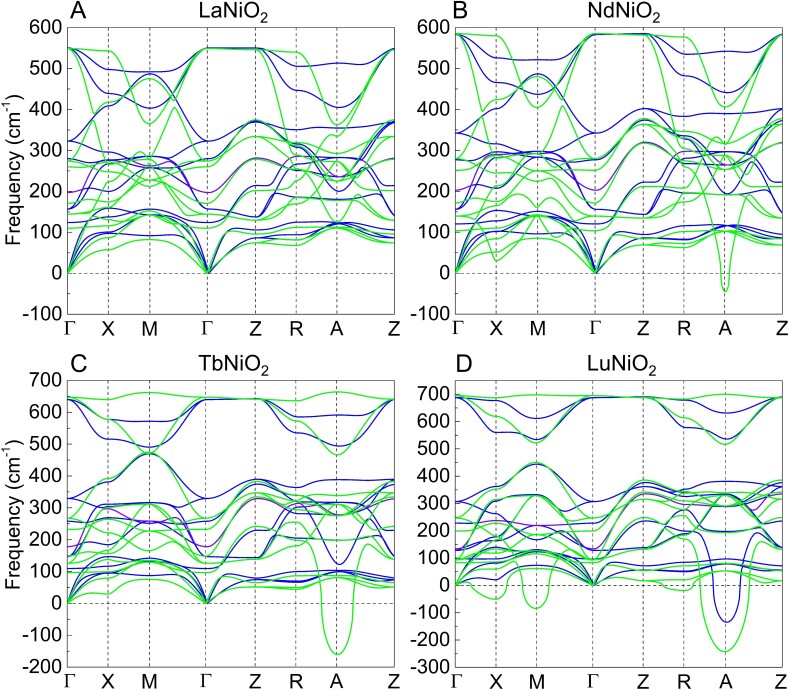
Dynamical properties of *R*NiO_2_ compounds. Phonon dispersion curves of A) LaNiO_2_, B) NdNiO_2_, C) TbNiO_2_, and D) LuNiO_2_ in their P4/mmm ground state magnetic phase and nonmagnetic phase. The blue curves and green curves represent the results from the nonmagnetic phase and magnetic phase, respectively. The high-symmetry points are denoted by: Γ=(0,0,0), X=(0,0.5,0), M=(0.5,0.5,0), Z=(0,0,0.5), R=(0,0.5,0.5), and A=(0.5,0.5,0.5). The unstable mode at high-symmetry A point is the out-of-phase rotation of the NiO_4_ square.

**Fig. 2.**
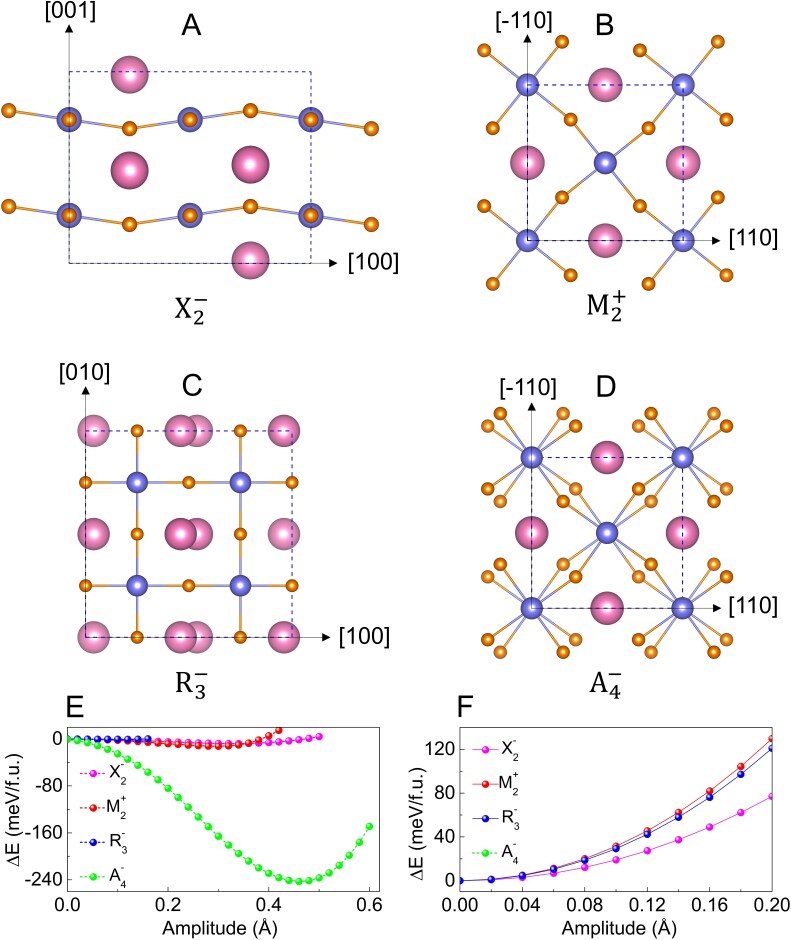
Unstable modes and lattice competition. Schematic pictures of the unstable modes in LuNiO_2_ including A) X${}_{2}^{-}$, B) M${}_{2}^{+}$, C) R${}_{3}^{-}$, and D) out-of-phase rotation A${}_{4}^{-}$. PESs of LuNiO_2_ along the lines of atomic displacements corresponding to E) the individual unstable modes at high-symmetry points and F) the same modes but with A${}_{4}^{-}$ mode condensed with its natural amplitude into the structure.

We start our study with a careful re-investigation of the structural properties of *R*NiO_2_ compounds (*R* = La, Pr, Nd, Sm, Eu, Gd, Tb, Dy, Ho, Er, Tm, and Lu), relaxing first their P4/mmm phase in A-AFM, C-AFM, ferromagnetic (FM), and G-AFM orders (see Fig. S1). To probe the dynamical stability of P4/mmm  *R*NiO_2_, we then calculated the phonon dispersion curves of LaNiO_2_, NdNiO_2_, TbNiO_2_, and LuNiO_2_ in both the magnetic and nonmagnetic phases. Surprisingly, although LaNiO_2_ appears as dynamically stable (see Fig. 1A), NdNiO_2_, TbNiO_2_, and LuNiO_2_ in their AFM state all show sizable phonon instabilities as illustrated in Fig. 1B to D. Moreover, decreasing the size of the *R*-site cation radius when going from NdNiO_2_ to LuNiO_2_, the number and amplitude of the instabilities progressively increase. The dominant instability is always an A${}_{4}^{-}$ mode associated with AFD out-of-phase rotation of the NiO_4_ squares around the c axis. The unstable modes in LuNiO_2_ are shown in Fig. 2A to D. The progressive destabilization of oxygen square rotation motion as the size of the *R*-site cation decreases is reminiscent of what is observed for the AFD motions of the oxygen octahedra in *R*NiO_3_ perovskites (50, 55) (Fig. S2). Importantly, we notice that previous calculations in the nonmagnetic state (27–29) missed the structural instability of NdNiO_2_ (as further confirmed here in Fig. 1B), while our present calculations reveal its presence when properly deals with the magnetic character recently confirmed experimentally (4), which reflects the spin-assisted instability of rotation distortion through the strong spin-rotation coupling.

In order to identify the ground-state structure of *R*NiO_2_ compounds, we fully relaxed various possible structures by condensing individual and combined phonon instabilities, taking NdNiO_2_ and LuNiO_2_ as prototypical examples. In both cases, the identified ground state is an I4/mcm phase obtained from the condensation of the A${}_{4}^{-}$ unstable mode of the P4/mmm phase.

For NdNiO_2_, this ground state is natural since A${}_{4}^{-}$ mode is the only phonon instability. For LuNiO_2_, the situation is more complicated as there are more unstable modes. However, the A${}_{4}^{-}$ instability remains dominant. As illustrated in Fig. 2E, the double-well potential energy surface (PES) associated with the A${}_{4}^{-}$ mode is significantly deeper than that related to other instabilities. Then, it is further clarified in Fig. 2F that, when condensing the A${}_{4}^{-}$ mode with its natural amplitude, other weaker instabilities disappear (i.e. all curves switch from double- to single-well shape). This reveals an inherent competition between out-of-phase rotation and other unstable modes: the appearance of the stronger rotation motion completely suppresses the other instabilities, stabilizing the I4/mcm structure as the ground state. Figure S3 shows a comparison between the energy of the possible phases in both the C-AFM and G-AFM states. It is obvious that a P4/mmm-I4/mcm structural phase transition with the appearance of $a^{0}a^{0}c^{-}$ rotation motion for *R* = Nd–Lu and a G-AFM-C-AFM magnetic transition occur simultaneously as the size of rare-earth ion decreases.

### Unusual upturn of resistivity

Having established that *R*NiO_2_ infinite-layer compounds are prone to AFD distortions, the natural question that arises concerning the temperature at which rotation appears. To access the finite-temperature behavior, we built a second-principles model (56) with the amplitudes of individual in-plane oxygen motion along the edges of NiO_4_ squares as degrees of freedom in the spirit of what was done by Zhong, Vanderbilt, and Rabe for perovskites (57). The model is directly fitted on first-principles data and finite-temperature properties are accessed from Monte Carlo simulations. A detailed description of the model is provided in the Method section.

**Fig. 3.**
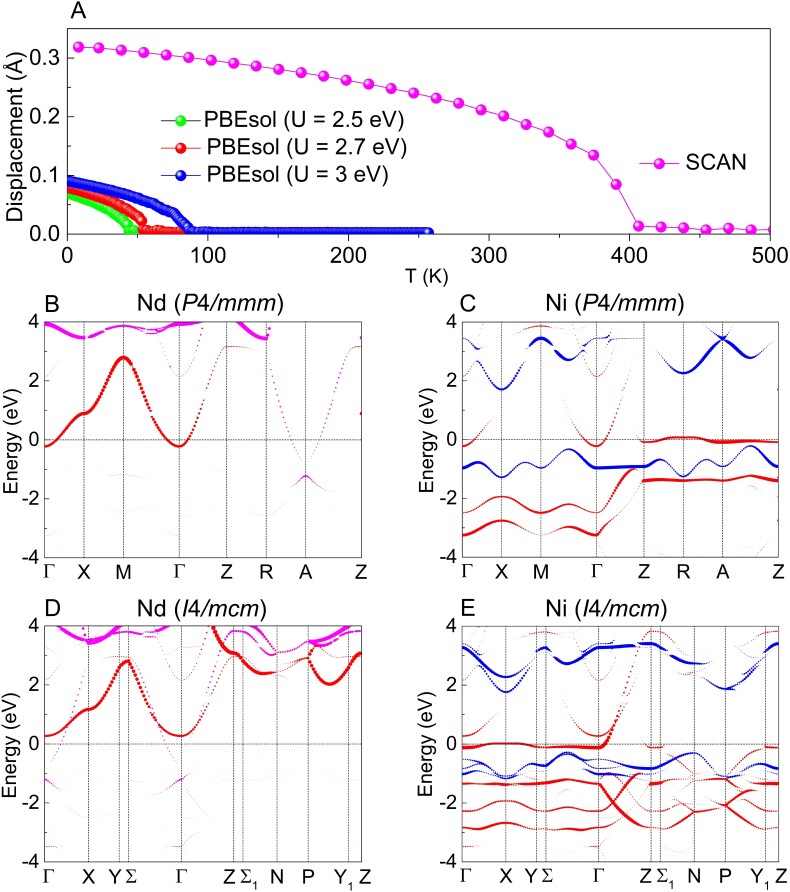
Finite-temperature properties of NdNiO_2_. A) Rotation motion induced displacement of oxygen atoms relative to the P4/mmm phase as a function of temperature obtained from PBEsol + U and SCAN methods. The orbital-projected band structure with orbital character of B) Nd $d_{xy}$ (magenta) and $d_{3z^{2}-r^{2}}$ (red) states and C) Ni $d_{3z^{2}-r^{2}}$ (red) and $d_{x^{2}-y^{2}}$ (blue) states in the C-AFM P4/mmm phase calculated by PBEsol + *U* (U=2.7 eV). The corresponding orbital-projected band structure of Nd and Ni atoms in the I4/mcm phase are shown in D) and E), respectively. The Fermi level denoted by the dash line is set to zero energy.

Results of the Monte Carlo simulations for NdNiO_2_ are reported in Fig. 3A that summarizes the temperature evolution of the average displacement associated to AFD oxygen rotation. The figure clearly highlights a structural phase transition from the high-symmetry P4/mmm phase to the low-symmetry I4/mcm phase. The displacive (58) or order-disorder (59) nature of the transition can be identified by anharmonic lattice-dynamics Hamiltonian (60) and probability distribution analysis (61). It is found that the phase transition temperature T_R_ from the revised Perdew–Becke–Erzenhof functional for solids (PBEsol) (62) + *U* (63) with U=2.5, 2.7, and 3 eV are very close to 70 K, after which the resistance begins to increase (1).

To verify whether the predicted structural phase transition is the key factor that leads to the unusual upturn of resistivity of NdNiO_2_ observed at low temperature (1) and shed light on the actual connection between temperature and electronic structure, the band structure of high-temperature P4/mmm phase and low-temperature I4/mcm phase are compared in Fig. 3B to E. Clearly, the band structures reflect the fact that the electronic structure at the Fermi level is very sensitive to the rotation distortion, thus confirming the dramatic influence of temperature. In detail, we found that although Nd $d_{xy}$ orbital is insensitive to temperature and rotation amplitude, the band edges of Nd $d_{3z^{2}-r^{2}}$ orbital and Ni $d_{3z^{2}-r^{2}}$ orbital at the Γ point of the band structure shift to an energy higher than the Fermi level, which significantly reduces the self-doping effect.

To quantify to which extent oxygen rotation suppressed self-doping effect can be a reliable explanation for the upturn of resistivity, a direct comparison of resistivity obtained from the Boltzmann transport equation (64) in the high-temperature P4/mmm phase and low-temperature I4/mcm phase is shown Fig. S4, it is clear that the resistivity around the Fermi level increases significantly in the low-temperature phase. Therefore, in line with superconducting LaOFeAs (47) and Sr_3_Rh_4_Sn${}_{13}$ (48), the resistivity anomaly observed at low temperature in NdNiO_2_ (1) and absent in LaNiO_2_ (dynamical stable in the magnetic P4/mmm phase) might be attributed to the structural phase transition. The results in Fig. 3A indicate that T_R_ gradually increases as *U* increases. As larger *U* values (4–5 eV) and the SCAN functional largely overestimate the transition temperature, a relatively small *U* of 2.7 eV is employed in our work.

### Rare-earth control of magnetic transition

**Fig. 4.**
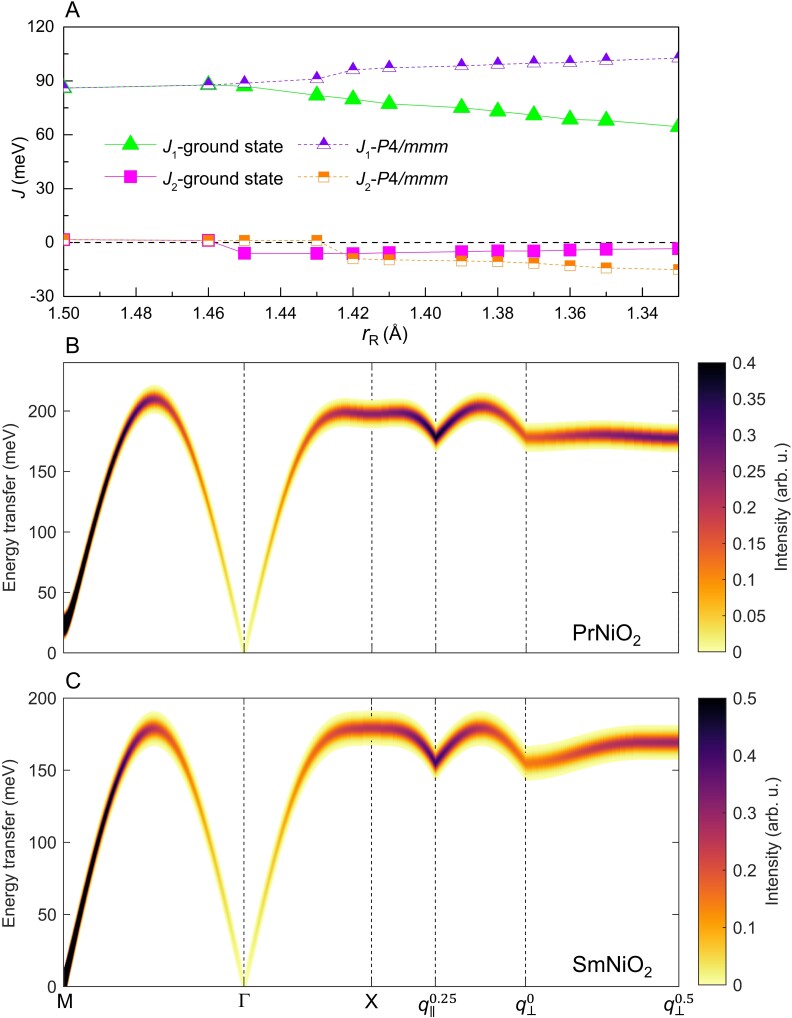
*R*-site cation determined magnetic properties of *R*NiO_2_. A) First-neighbor in-plane ($J_{1}$) and out-of-plane ($J_{2}$) exchange constants for different *R*NiO_2_ compounds in the P4/mmm and I4/mcm phases. The simulated adiabatic spin-wave dispersions of B) PrNiO_2_ and C) SmNiO_2_. Here, M=(0.5,0.5,0), Γ=(0,0,0), X=(0.5,0,0), $q_{\|}^{0.25}=(0.5,0.25,0)$, $q_{⊥}^{0}=(0.25,0,0)$, and $q_{⊥}^{0.5}=(0.25,0,0.5)$.

Figure 4A shows the variation of first-neighbor exchange constants across the *R*NiO_2_ series in the ground state phases and high-symmetry P4/mmm phase. In terms of first-neighbor out-of-plane exchange constant, we see an abrupt change around the phase boundary in the ground state phases, LaNiO_2_ and PrNiO_2_ exhibit negligible out-of-plane magnetic coupling, similar with cuprate superconductors (65). In contrast, *R*NiO_2_ with *R* = Nd–Lu possess the 3D magnetic interactions with nonnegligible out-of-plane FM magnetic coupling, similar with iron superconductors (66–68). In order to confirm how the rare-earth ion controls the magnetic dimensionality, the magnetic excitation dispersion of PrNiO_2_ and SmNiO_2_ are compared in Fig. 4B and C, respectively. The spectra of PrNiO_2_ exhibits similar characters as the recent resonant inelastic X-ray scattering (RIXS) experiment (40), indicating that the employed long-range magnetic order is a good approximation of the experimental results. The negligible and nonnegligible dispersion along the out-of-plane (0.25, 0, 0)–(0.25, 0, 0.5) path (measured in the RIXS experiment of NdNiO_2_ (4)) in PrNiO_2_ and SmNiO_2_ imply their quasi-2D AFM and 3D-AFM nature. Consequently, rare-earth ion not only controls the magnetic order, but also the magnetic dimensionality of *R*NiO_2_.

### Spin-rotation coupling determined in-plane exchange constants

It is generally believed that superconductivity in iron and cuprate superconductors is related to the in-plane AFM coupling. Moreover, the rare-earth element is a key factor affecting the superconducting temperature (69, 70). It is thus important to evaluate the variation of dominant exchange constants across the *R*NiO_2_ series.

Typically, the magnetic coupling in ABX_3_ perovskites strongly depends on the variation of lattice constant and rotation angle (71): when the radius of A-site cation decreases, the lattice constants decrease while the rotation angle gradually increases. The reduction of the lattice constants typically enhances the magnetic coupling, while the increase in rotation weakens the magnetic interaction (71), thus, the actual influence of *A*-site cation on the exchange constants is determined by the competing effects of lattice constants and rotation. In *R*NiO_2_, the decrease in the *R*-site cation radius also gives rise to a decrease in the lattice constants and an increase in rotation in a similar way as ABX_3_ perovskites (71). It can thus be expected that taking only into account the influence of lattice constant evolution on the exchange coupling, as considered in previous works (19, 23), is not enough to describe the global effect of *R*-site cation substitution: the additional effect of oxygen rotation amplitudes must be taken into account.

Figure 4A also displays the exchange constants in the high-symmetry P4/mmm phases without rotation, we found that the in-plane first-neighbor magnetic coupling is progressively increased when decreasing $r_{R}$, similar to recent work (23). In contrast, we obtain an almost opposite trend for the in-plane AFM coupling in the ground state I4/mcm phase compared with (23) as it gradually decreases as $r_{R}$ decreases when *R* = Nd–Lu as shown in Fig. 4A. Therefore, the decreased in-plane AFM coupling for smaller $r_{R}$ in the ground state I4/mcm phase should be attributed to the increase in rotation, whose effect is partly compensated by the lattice constants effect. The $r_{R}$ dependent AFM coupling is completely in line with rare-earth perovskites like *R*CrO_3_ (72), *R*FeO_3_ (73), and *R*NiO_3_ (74), indicating that rare-earth ions in infinite-layer nickelates affect the magnetic interactions through spin-rotation coupling.

### Rare-earth control of electronic transition

**Fig. 5.**
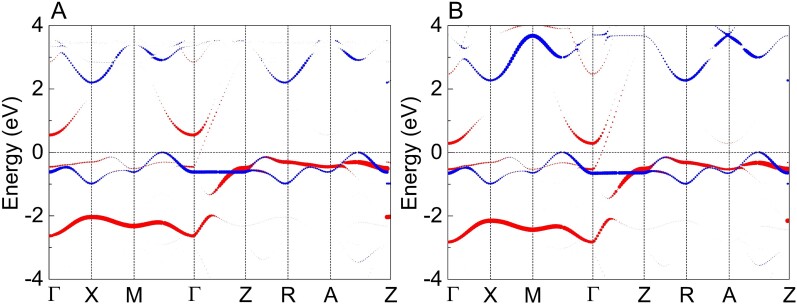
Electronic structure in quasi-2D AFM *R*NiO_2_. The orbital-projected band structure with orbital character of Ni $d_{3z^{2}-r^{2}}$ (red) and $d_{x^{2}-y^{2}}$ (blue) states for A) LaNiO_2_ and B) PrNiO_2_.

To account for the electronic origin of magnetic transition in *R*NiO_2_, we then compare the orbital-projected band structure of LaNiO_2_ and PrNiO_2_ in Fig. 5 with that of NdNiO_2_ in Fig. 3E. Strikingly, there is a sudden reconstruction of the Fermi surface coinciding with the magnetic transition. The decrease in $r_{R}$ from La(Pr)NiO_2_ to NdNiO_2_ shifts the band edge of $d_{3z^{2}-r^{2}}$ orbital to a higher energy than the Fermi level. The dominated states at the Fermi level have changed from $d_{x^{2}-y^{2}}$ bands to $d_{3z^{2}-r^{2}}$ bands, which is characteristic of an electronic transition. The electronic structure of NdNiO_2_ is in line with previous works (19, 20, 21, 22, 75) and reminiscent of the orbital-selective localization as found in alkaline iron selenides (76, 77) and (Ca,Sr)_2_RuO_4_ (78, 79), which is also suggested in previous works (9–12, 80).

Table 1 compares the orbital contributed first-neighbor exchange constants among LaNiO_2_, PrNiO_2_, NdNiO_2_, and SmNiO_2_. The predicted exchange constants for PrNiO_2_ and NdNiO_2_ are very close to the experimental results (4, 40). Moreover, the first-neighbor in-plane exchange constants in different compounds are all mainly contributed by the interactions between nearest-neighbor Ni $d_{x^{2}-y^{2}}$ orbitals, whereas the Ni $d_{3z^{2}-r^{2}}$ orbital is responsible for the out-of-plane FM coupling in NdNiO_2_ and SmNiO_2_. Consequently, the quasi-2D and 3D magnetic dimensionality in La(Pr)NiO_2_ and Nd(Sm)NiO_2_ lies in the absence and presence of spin-polarized $d_{3z^{2}-r^{2}}$ electrons at the Fermi level.

**Table 1.** First-neighbor exchange constants and their orbital contributions for LaNiO_2_, PrNiO_2_, NdNiO_2_, and SmNiO_2_ in the unit of meV. The dominant contributions to the first-neighbor in-plane magnetic interactions are labeled in bold.

	Total	$d_{x^{2}-y^{2}}-d_{x^{2}-y^{2}}$	$d_{x^{2}-y^{2}}-d_{xy}$	$d_{3z^{2}-r^{2}}-d_{3z^{2}-r^{2}}$	$d_{x^{2}-y^{2}}-d_{3z^{2}-r^{2}}$	$d_{xy}-d_{xy}$	$d_{xz}-d_{xz}$	$d_{yz}-d_{yz}$
J_1_ (LaNiO_2_)	85.98	**85.80**	0.00	0.10	0.16	0.00	0.06	−0.14
J_1_ (PrNiO_2_)	87.24	**87.05**	0.00	0.20	0.11	0.00	0.08	−0.20
J_1_ (NdNiO_2_)	87.07	**82.93**	2.98	0.29	0.74	0.10	0.06	−0.03
J_2_ ((NdNiO_2_)	−5.98	−0.26	−0.02	** −6.10 **	0.00	0.04	0.18	0.18
J_1_ (SmNiO_2_)	82.03	**74.11**	6.67	0.34	0.59	0.29	0.05	−0.02
J_2_ ((SmNiO_2_)	−6.01	−0.17	−0.04	** −6.16 **	0.00	0.04	0.16	0.16

### Rare-earth control of upper critical magnetic

The unexpected isotropy of the upper critical magnetic field H${}_{c2}$ is recently revealed in experiments (5, 6, 39). Ferropnictides are multiband superconductors with both Mott-localized and itinerant electrons (66–68, 81, 82), similar with 3D C-AFM NdNiO_2_. It has been revealed that $k_{z}$ dispersion naturally facilitates the circulating currents at all field orientations and results in the isotropic H${}_{c2}$ as found in ferropnictides (46). Interestingly, the band structure of NdNiO_2_ shown in Fig. 3E shows a strong $k_{z}$ dispersion denoted by the large weight of the Ni $d_{3z^{2}-r^{2}}$ band at the Fermi level. In contrast, there is rather weak $k_{z}$ dispersion at the Fermi level for LaNiO_2_ (Fig. 5A) and PrNiO_2_ (Fig. 5B) as reflected by the small weight of the Ni $d_{3z^{2}-r^{2}}$ band. Therefore, our results support the fact that the strength of $k_{z}$ dispersion in NdNiO_2_ and La(Pr)NiO_2_ are likely responsible for their isotropic and anisotropic H${}_{c2}$ observed in experiments.

### Structural origin of magnetic and electronic transitions

**Fig. 6.**
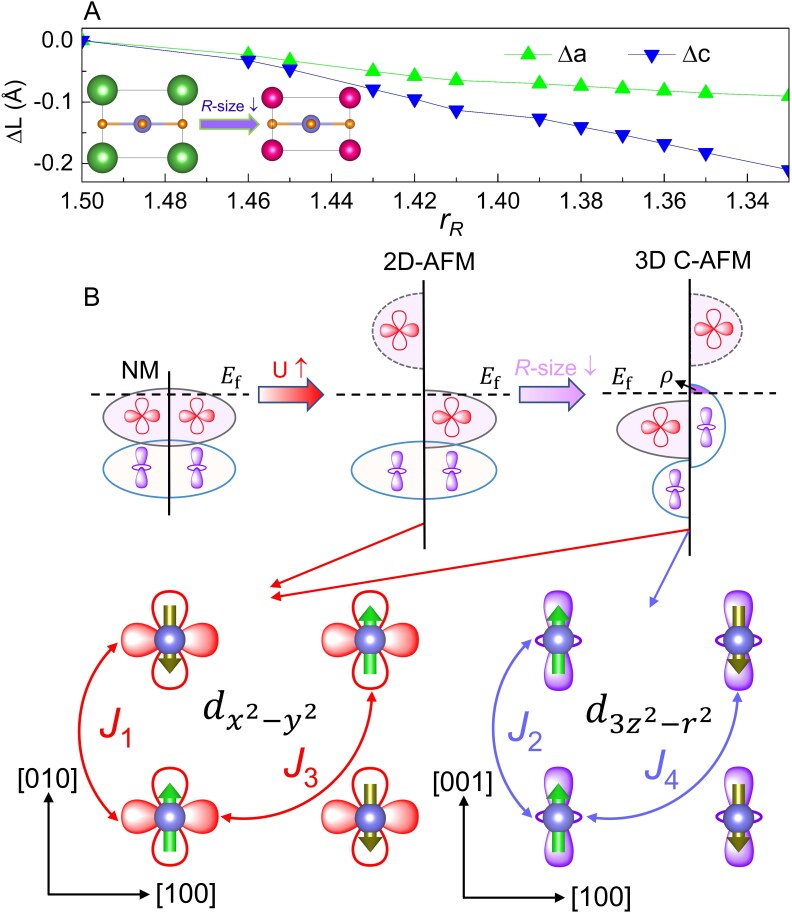
Structural origin of magnetic and electronic transitions. A) Evolution of in-plane and out-of-plane lattice constants of *R*NiO_2_ relative to LaNiO_2_. The inset atomic structures denote that the reduction of rare-earth size decreases the c/a ratio, which is analogous to the effect of epitaxial biaxial tensile strain. B) Schematic of the interplay among lattice, electron, and spin degrees of freedom. $E_{f}$, Fermi level; U, Hubbard U value of *d* electron; ρ, the unoccupied $d_{3z^{2}-r^{2}}$ state; ↑ and ↓ denote increase and decrease, respectively.

In order to disentangle the microscopic mechanism behind the *R*-site cation controlled electronic and magnetic transitions, we focus on the structural effect induced by rare-earth ion. The variation of in-plane and out-of-plane lattice constants are compared in Fig. 6A. As $r_{R}$ decreases, despite the in-plane and out-of-plane lattice constants both progressively decrease, the reduction of out-of-plane lattice constant is much faster than that of in-plane lattice constants consistent with previous work (23). Thus, the decrease in rare-earth ionic radius reduces the *c*/*a* ratio analogous to that of epitaxial biaxial tensile strain.

From the above analysis, we notice that the main difference between 2D-AFM La(Pr)NiO_2_ and 3D C-AFM Nd(Sm)NiO_2_ arises from the itinerant $d_{3z^{2}-r^{2}}$ bands, which is spin-polarized and has a higher energy level than the $d_{x^{2}-y^{2}}$ bands in the 3D C-AFM state. In ABX_3_ perovskites, epitaxial biaxial tensile strain directly yields the elongation and contraction of the in-plane and out-of-plane lattice constant, respectively. Such effect typically weakens and strengthens the hybridization of $d_{x^{2}-y^{2}}$ and $d_{3z^{2}-r^{2}}$ bands with the surrounding O *p* orbitals. As a result, the on-site energy of $d_{x^{2}-y^{2}}$ and $d_{3z^{2}-r^{2}}$ orbitals will shift to a lower and higher energy level, respectively (83).

The strain-orbital relationship in perovskites could be naturally extended to infinite-layer nickelates and is indispensable to rationalize the *R*-site cation-tuned electronic and magnetic transitions. The evolution of electronic structure and direct structure–electron–magnetism relationship are schematically shown in Figs. 6B and S5.

In the nonmagnetic state, the out-of-plane $d_{3z^{2}-r^{2}}$ state is lower than the in-plane $d_{x^{2}-y^{2}}$ state due to the crystal field, and is almost fully occupied by a pair of electrons (also see Fig. S5A). The reduction of the bandwidth of partly occupied $d_{x^{2}-y^{2}}$ bands by the electron–electron interaction *U* gives rise to a spin Mott splitting, which shifts the majority and minority spin channel below and above the Fermi energy for *R* = La–Pr (also see Fig. S5B). The spin splitting of $d_{x^{2}-y^{2}}$ bands at the Fermi surface results in the 2D-AFM state with mainly in-plane magnetic interactions. As the reduction of the rare-earth size gradually decreases the *c*/*a* ratio, the hybridization between the interlayer Ni $d_{3z^{2}-r^{2}}$ orbital and their on-site energy is increased. Consequently, the Ni $d_{3z^{2}-r^{2}}$ bands become spin polarized and occupy the Fermi level for *R* = Nd–Lu. (also see Fig. S5C) Since the spin-polarized $d_{3z^{2}-r^{2}}$ electrons are decisive for the out-of-plane FM magnetic coupling (also see Table 1), the geometrical effect controlled electronic transition is correspondingly accompanied by the change in magnetic dimensionality. The results are based on the density functional theory (DFT) + *U* ground state calculations, to further investigate the electronic structure in high-temperature PM phase and quantum many-body effects, dynamical mean field theory (DMFT) (14–17, 13, 18) method should be employed.

### Phase diagram of *R*NiO_2_ compounds

**Fig. 7.**
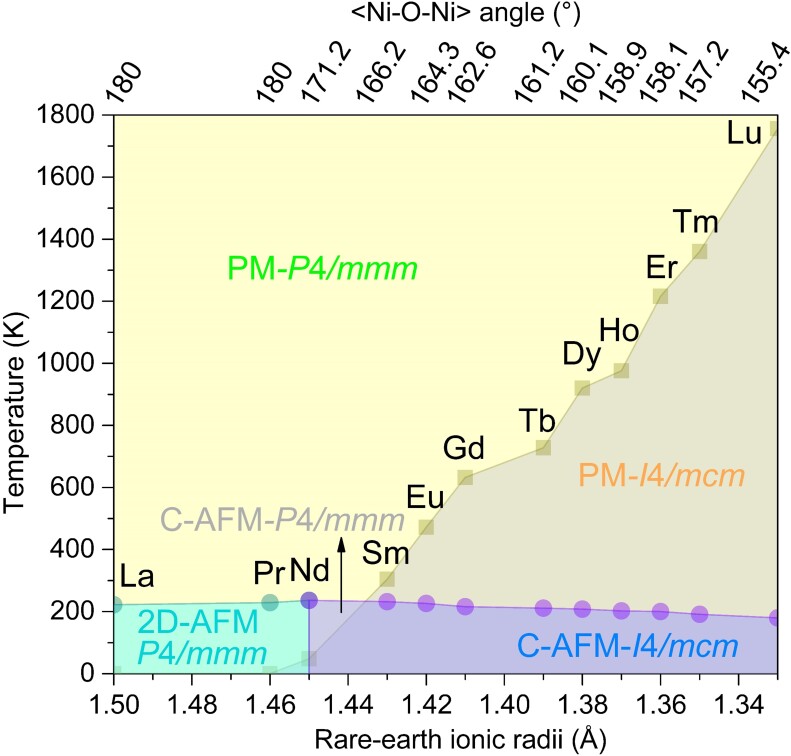
Phase diagram of infinite-layer nickelates in terms of rare-earth ionic radii and the temperature with PBEsol + *U* (U=2.7 eV). The circles refer to the AFM–PM transition temperature and the squares represent the transition temperature from the I4/mcm phase to the P4/mmm phase.

Combining the Néel temperature obtained from the Monte Carlo simulations of the Heisenberg spin Hamiltonian and structural transition temperature determined by the second-principles model, the temperature-dependent structural and magnetic transitions of the whole family of *R*NiO_2_ compounds are summarized in the global phase diagram reported in Fig. 7. Except for high-symmetry LaNiO_2_ and PrNiO_2_, we see that all the other infinite-layer nickelate compounds undergo a structural phase transition from the low-symmetry I4/mcm phase to the high-symmetry P4/mmm phase. Moreover, as rare-earth ionic radii $r_{R}$ decreases, the transition temperature T_R_ continuously increases from NdNiO_2_ to LuNiO_2_ due to the more distorted structure, as similarly observed in *R*NiO_3_ perovskites (50).

Owning to the decreased in-plane first-neighbor exchange constant from NdNiO_2_ to LuNiO_2_, it is found that the AFM–PM transition temperature T_N_ decreases steadily as $r_{R}$ decreases (except the high-symmetry LaNiO_2_ and PrNiO_2_) which is also strongly analogous to the behavior observed in *R*NiO_3_ (*R* = Sm–Lu) perovskites (84). Consequently, the presence of rotation motion not only affects the electronic property and the overall magnitude of the magnetic transition temperature, but gives rise to a much more complex phase diagram with five distinct phases: (i) the 2D-AFM high-symmetry P4/mmm phase, (ii) the C-AFM low-symmetry I4/mcm phase, (iii) the C-AFM high-symmetry P4/mmm phase, (iv) the PM low-symmetry I4/mcm phase, and (v) the PM high-symmetry P4/mmm phase. The complex phase diagram of infinite-layer nickelates shows strong similarities with the phase diagram of perovskite nickelates and the dedicated interplay among the spin, electron, rotation, and temperature opens new perspectives for the control of magnetic and superconducting properties by exploiting the different strategies previously used in perovskite nickelates.

## Conclusion and outlook

In summary, we present a systematic theoretical investigation of the effect of rare-earth ions by explicitly exploring the interplay among the structural, electronic, and magnetic degrees of freedom in infinite-layer nickelates. Structurally, we reveal that oxygen rotation distortion is destabilized with reducing rare-earth size and strengthened by magnetism, which triggers a phase transition from P4/mmm to I4/mcm at low temperature for *R*NiO_2_ (*R* = Nd–Lu). The rotation motion appears to be the key to provide a consistent description of the structural, electronic, and magnetic properties of infinite layers. First, it strongly suppresses the self-doping effect, which can explain the unusual upturn of resistivity in NdNiO_2_ observed at low temperature (1). Second, the rotation has to be included to provide a proper description of the evolution of the magnetic coupling strength in *R*NiO_2_.

From electronic and magnetic viewpoints, reducing the rare-earth size plays a similar role as epitaxial tensile strain, which strongly affects the competition between localized Ni $d_{x^{2}-y^{2}}$ electrons and itinerant Ni $d_{3z^{2}-r^{2}}$ electrons, magnetic coupling strength, and magnetic dimensionality, resulting in two possible ground states. Compounds with larger *c*/*a* ratio such as LaNiO_2_ and PrNiO_2_ resemble the key electronic and magnetic properties of CaCuO_2_. However, compounds with smaller *c*/*a* ratio (*R* = Nd–Lu) show marked electronic and magnetic resemblance to ferropnictides with notable interlayer FM coupling and strong $k_{z}$ dispersion, which may help elucidate the small anisotropy of the upper critical field of NdNiO_2_ observed experimentally.

Based on the actual interplay among different factors, we eventually build a global and clear theoretical description of the lattice–electron–spin relationship. Accordingly, a complete phase diagram summarizing the temperature evolution of structural and magnetic phases in the whole family of *R*NiO_2_ compounds is established. Our work not only provides a deeper fundamental understanding of *R*NiO_2_ compounds that explicitly combines the interplay among different degrees of freedom, but also exploits this knowledge to explain the rare-earth control of resistivity and upper critical magnetic field observed in experiments. As such, we hope that our work could motivate experimentalists to further exploit the coupling among rotation, charge, orbital, spin, and strain degrees of freedom in infinite layer *R*NiO_2_ to realize the optimization of the applications as superconductors.

## Methods

### First-principles calculations

Our DFT plus *U* (U=2.7 eV) calculations (63) were carried out using the projector augmented wave (PAW) method implemented in Vienna ab initio simulation package (VASP) (85, 86). The revised Perdew–Becke–Erzenhof functional for solids (PBEsol) (62) was employed. For the structural relaxation, the energy and Hellmann–Feynman forces tolerance were set to be $10^{-7}$ eV and $10^{-3}$ eV/Å, respectively. The kinetic energy cutoff of the plane-wave basis was 700 eV and the Brillouin-zone integrations were sampled by 9×9×7 Γ-centered k-point mesh (87). The phonon dispersion calculations were performed using the finite displacement method as implemented in the PHONOPY code (88).

### Second-principles calculation

First-principles based second-principles method is applied to investigate the evolution of rotation motion at different temperatures. We build a second-principles model with displacements of oxygen atom *μ* as the degree of freedom. The total energy can be expressed as


(1) $$E_{\mathrm{total}}=E_{\mathrm{self}}+E_{\mathrm{short}}$$

Where $E(\mu_{i})$ in the first term $E_{\mathrm{self}}=\sum_{i}E(\mu_{i})$ represents the energy of an isolated oxygen atom at *i*th location with amplitude $\mu_{i}$. It is truncated at fourth order, and the maximum energy difference between our model and DFT is smaller than 1 meV per unit cell, which indicates this model is good enough to describe the PESs. Due to the symmetry consideration, $E(\mu_{i})$ can be written as


(2) $$E(\mu_{i})=k_{1}\mu_{i}^{2}+k_{2}\mu_{i}^{4}$$

Where $k_{1}$ and $k_{2}$ refer to the parameters to be determined from first-principles calculations by fitting the total energy of structures with eight different modes shown in Fig. S6A to P. In the second term $E_{\mathrm{short}}=\sum_{i,j}g_{ij}\mu_{i}\mu_{j}$, $\mu_{i}$ and $\mu_{j}$ are the amplitude of oxygen atom displacements at *i*th and *j*th location and $g_{ij}$ is the coupling parameter between them. This term is the energy contribution from the short-range interactions between neighboring oxygen atoms. The length of short-range interactions is truncated at one unit cell. Due to the symmetry consideration, there are only four independent interaction parameters ($g_{1}$, $g_{2}$, $g_{3}$, and $g_{4}$) for the short-range interactions as sketched in Fig. S6Q and R. All these parameters are obtained from first-principles calculations. Based on the second-principles model, the Monte Carlo simulations are carried out to investigate the rotation motion with a heating run from 0 to 2000 K in steps of 1 K. For each temperature steps, 100,000 Monte Carlo steps are performed. The calculations were performed on a 12 ×12×12 supercell. To guarantee an acceptance ratio of 0.2, the step sizes are adjusted accordingly. The total energies obtained from first-principles calculations and second-principles model are compared in Fig. S6S. The close match of the energies guarantees the accuracy and validity of our model.

### Exchange constant and magnetic excitations calculations

The exchange constants and the orbital contributions were calculated by using the TB2J code (89). In this approach, magnetic force theorem (90) is implemented based on the maximally localized Wannier functions (91, 92), which were built from the DFT band structure. The Nd d${}_{xy}$ and $d_{3z^{2}-r^{2}}$ orbitals, Ni $d_{xy}$, $d_{yz}$, $d_{zx}$, $d_{x^{2}-y^{2}}$, and $d_{3z^{2}-r^{2}}$ orbitals and O $p_{x}$, $p_{y}$, and $p_{z}$ orbitals are used for constructing the Wannier functions. The Heisenberg Hamiltonian in the TB2J code is


(3) $$H=\sum_{\left\langle i,j\right\rangle }J_{ij}S_{i}S_{\;j}$$

where $J_{ij}$ denotes the exchange constant at any order and S=1/2 spin. The spin-wave spectra is calculated by the SpinW program (93) based on the obtained exchange constants.

## Supplementary Material

pgad108_Supplementary_Data

## Data Availability

All data are contained within the manuscript.
