# Supplementary material for: Rare-earth control of phase transitions in infinite-layer nickelates
Source: PNAS Nexus. 2023 Mar 29;2(5):pgad108. doi: 10.1093/pnasnexus/pgad108 (PMC10167552; doi:10.1093/pnasnexus/pgad108)
Supplement: pgad108_Supplementary_Data [file pgad108_supplementary_data.docx]

**
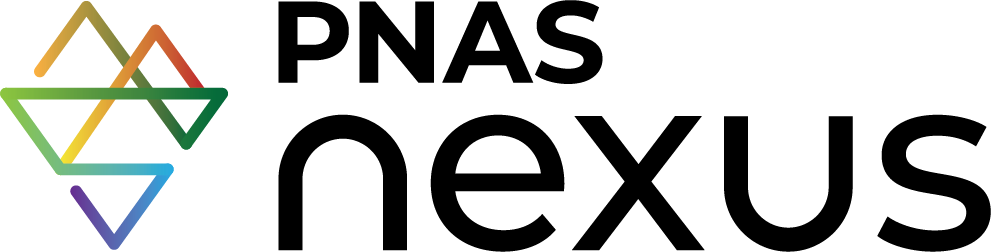
**

**Supplementary Information for**

**Rare-earth control of phase transitions in infinite-layer nickelates**

Yajun Zhang,^a,b,*,1^ Jingtong Zhang,^c,d,e,1^ Xu He,^c^ Jie Wang,^d,e^ and Philippe Ghosez^c^

^a^Key Laboratory of Mechanics on Disaster and Environment in Western China, Ministry of Education of China, Lanzhou University, Lanzhou, Gansu 730000, China

^b^Department of Mechanics and Engineering Sciences, College of Civil Engineering and Mechanics, Lanzhou University, Lanzhou, Gansu 730000, China

^c^Theoretical Materials Physics, Q-MAT, CESAM, Université de Liége, B-4000 Liége, Belgium

^d^Department of Engineering Mechanics and Key Laboratory of Soft Machines and Smart Devices of Zhejiang Province, Zhejiang University, 38 Zheda Road, Hangzhou 310027, China

^e^Zhejiang Laboratory, Hangzhou 311100, Zhejiang, China

Corresponding author: Yajun Zhang

Email: [zhangyajun@lzu.edu.cn](mailto:zhangyajun@lzu.edu.cn)

**This PDF file includes:**

Supplementary text

Figures S1 to S6





**Fig. S1.** Schematic pictures of *P*4*/mmm* *R*NiO_2_ with (A) A-AFM, (B) C-AFM, (C) FM, and (D) G-AFM orders.


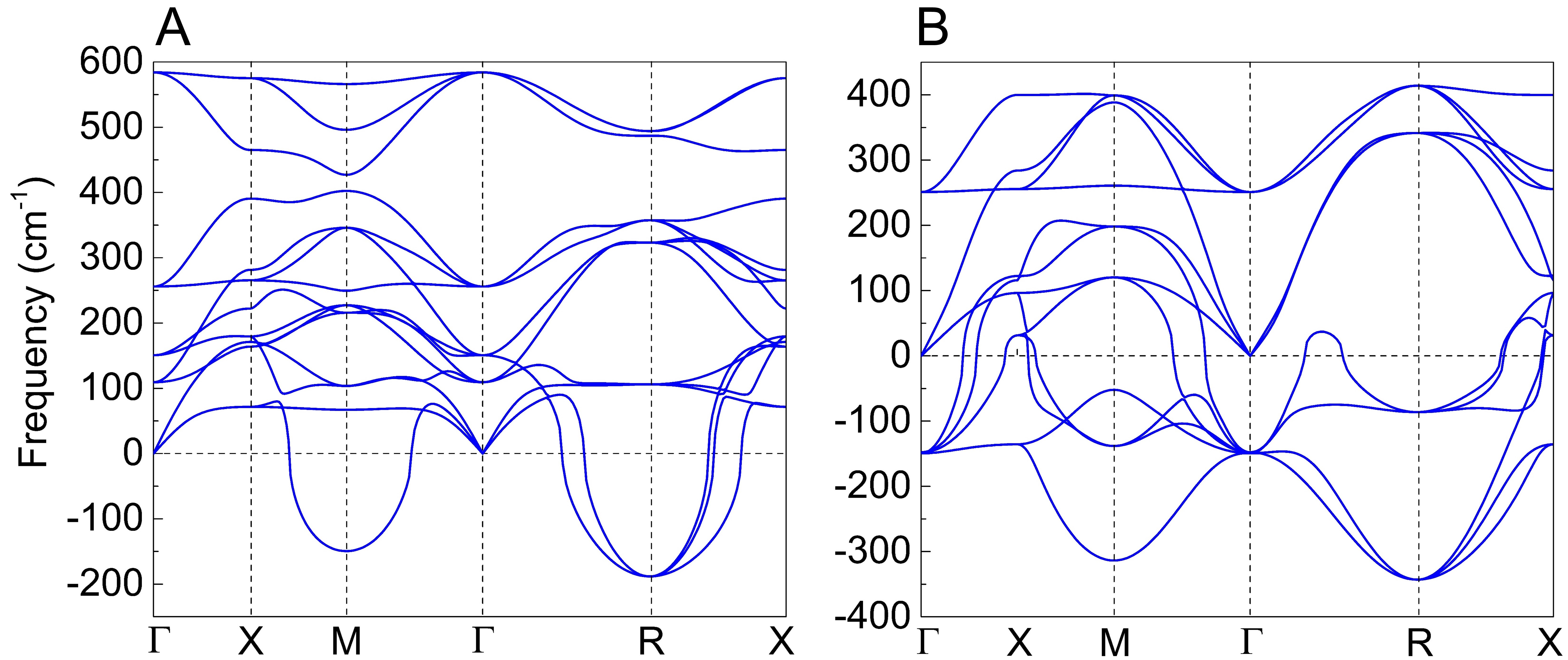


**Fig. S2.** Phonon dispersions of the FM *Pm*$\bar{3}$*m* (A) LaNiO_3_ and (B) LuNiO_3_.





**Fig. S3.** Energy difference of different phases relative to the reference G-AFM *P*4*/mmm* phase for *R*NiO_2_.





**Fig. S4.** Resistivity of the high-temperature *P*4*/mmm* (red curve) and low-temperature *I*4*/mcm* (green curve) NdNiO_2_ as determined from the Boltzmann transport equation.





**Fig. S5.** Projected density of states (PDOS) for (A) non-magnetic (NM) LaNiO_2_ obtained from PBEsol functional, (B) 2D-AFM LaNiO_2_ obtained from PBEsol +U (U = 2.7 eV), and (C) C-AFM SmNiO_2_ obtained from PBEsol +U (U = 2.7 eV).


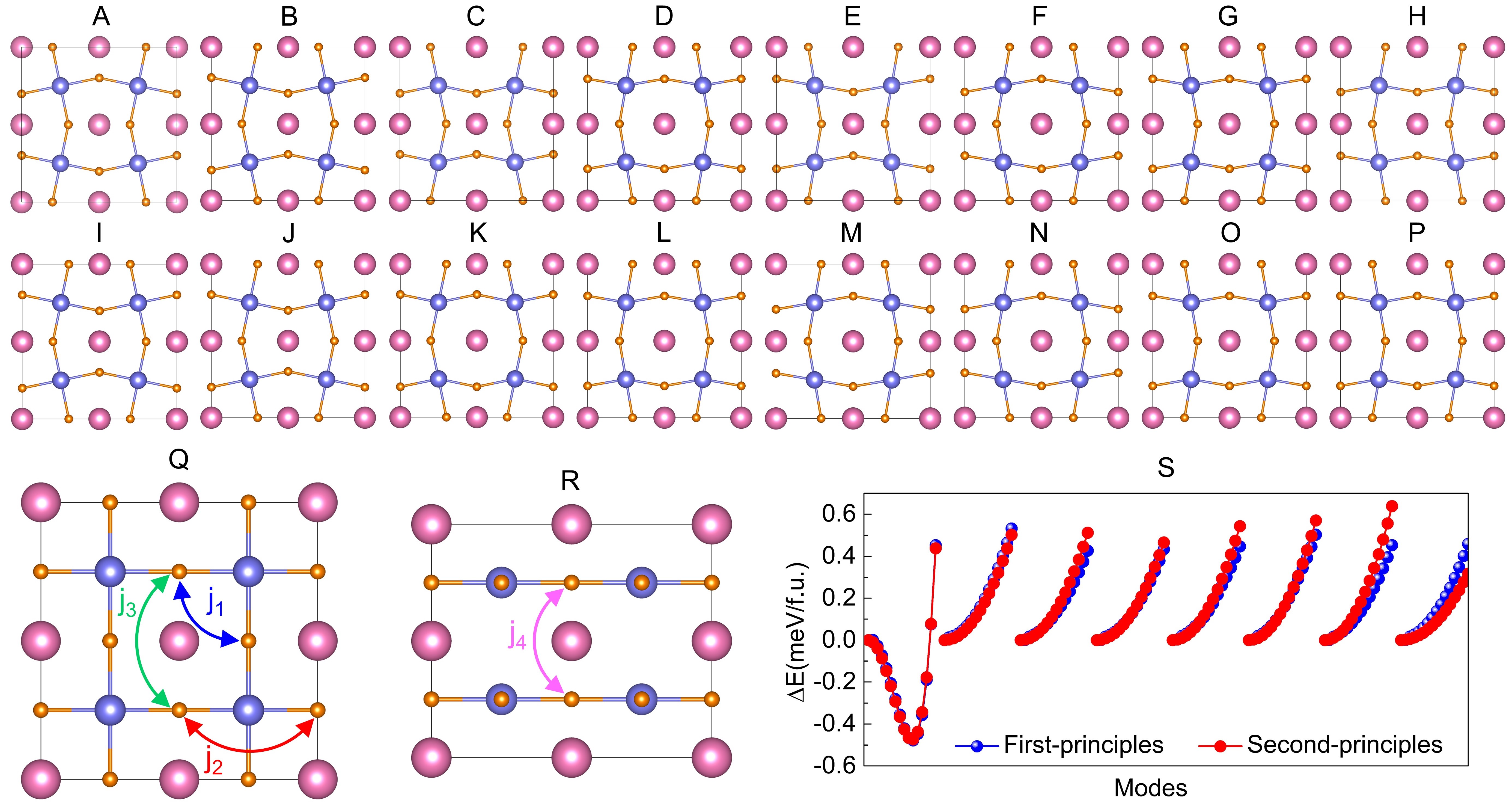


**Fig. S6.** (A)-(P) Schematic pictures of eight structures in the 2 × 2 × 2 supercells for fitting parameters in the second-principles model, Figures S6 (A)-(H) show the distortions in the first NiO_4_ layer and Figs. S6 (I)-(P) show the distortions in the second NiO_4_ layer. The distortions in the first structure (Fig. S6(A) and S6(I)) are the unstable out-of-phase rotation. (Q)-(R) Schematic representation of four short-range interactions used in the second-principles model, (S) the comparison of the energy evolutions between first-principles calculations and second-principles model for eight structures with different modes.
